# Clinical characteristics and optical coherence tomography of concomitant macular hole and rhgematogenous retinal detachment

**DOI:** 10.1038/s41598-024-61899-2

**Published:** 2024-05-26

**Authors:** Benjamin Chi-Lan Yang, Mei-Chi Tsui, Chung-May Yang, Yu-Teng Fu, Jiunn-Feng Hwang, Tso-Ting Lai, Tzyy-Chang Ho, Chang-Ho Yang, Yi-Ting Hsieh, San-Ni Chen

**Affiliations:** 1 Department of Ophthalmology, Eye Center, China Medical University Hospital, No. 91, Hsueh Shih Road, Taichung, 404 Taiwan; 2 Department of Ophthalmology, Changhua Christian Hospital, Changhua City, Taiwan; 3 Department of Ophthalmology, National Taiwan University Hospital, 7, Chung Shan South Road, Taipei, Taiwan; 4 Department of Ophthalmology, College of Medicine, National Taiwan University, Taipei, Taiwan; 5 Department of Ophthalmology, China Medical University, Taichung, Taiwan

**Keywords:** Macular hole, Rhegmatogenous retinal detachment, Optical coherence tomography, Choroid detachment, Ellipsoid zone lining in macular hole, Retina, Eye diseases

## Abstract

To study the clinical characteristics of macula off rhegmatogenous retinal detachment (RRD) with peripheral causative breaks and concomitant macular hole (RRD+MH). This is a bi-center study. Consecutive eyes of macula off RRD with or without macular hole (MH) were collected. Eyes in these two groups were compared with best corrected visual acuity in logarithm of minimal angle of resolution (logMAR BCVA), the presence of choroidal detachment (CD), proliferative vitreoretinopathy (PVR) and the extent of RRD. In the group of RRD+MH, regression analysis was used to evaluate the correlation of clinical factors and final logMar BCVA. In addition, optical coherence tomography was performed both pre-and post-operatively if possible. There were 40 eyes in the RRD+MH group and 80 eyes in the control group. Eyes with RRD+MH had worse initial and final logMar BCVA (p < 0.001), higher incidence of CD (p < 0.001), PVR and extensive RRD at baseline (p < 0.001). Among the eyes with RRD+MH, final BCVA was correlated with initial BCVA (p < 0.001, CI 0.637 to 0.837), recurrent RRD (p = 0.004, CI − 0.661 to − 0.126), duration of RRD (p = 0.021, CI − 0.576 to − 0.048) and presence of PVR (p = 0.001, CI − 0.131 to − 0.035). The hole closure rate at final follow up is 87.5%.11 of the 17 eyes had preoperative optical coherence tomography (OCT) obtained had ellipsoid zone lining the bottom of MH. CD, PVR and extensive RRD were more commonly observed in RRD+MH. The morphology of MH may suggest the pathogenesis of MH in RRD+MH include mechanism different from that of idiopathic MH.

## Introduction

Macular hole coexisting with peripheral break(s) induced rhegmatogenous retinal detachment (RRD+MH) is noted in 1 to 3% of patients^1–5^.The surgical goal has always been to reattach the retina; however, the techniques have evolved from treating the peripheral break(s) only by scleral buckling or vitrectomy to addressing both the peripheral beak(s) and the concomitant macular hole (MH) with vitrectomy and internal limiting membrane (ILM) peeling with or without ILM flap^1,2,4^. Previous reports showed that patients with RRD+MH had higher chance of proliferative vitreoretinopathy (PVR)^1^, higher reoperation rate and less favorable visual outcome, compared to patients with simple RRD without MH^2^. The pathogenesis of idiopathic macular hole has been well established in previous studies; however, the nature of macular hole in RRD has not been elucidated. In this study, we aim to study the characteristics and risk factors of RRD+MH, the morphology of the MH from pre-operative optical coherence tomography (OCT) and the possible pathogenesis of MH formation.

## Material and methods

This is a retrospective, comparative, bi-center study in Changhua Christian Hospital and National Taiwan University Hospital. The study protocol was adhered to the tenets of the declaration of Helsinki and was approved by the Institutional Review Board for Human Subjects Research at Changhua Christian Hospital and National Taiwan University Hospital. Retrospective chart review was performed on patients with primary rhegmatogenous retinal detachment (RRD) and had vitrectomy as the primary treatment. Consecutive patients of RRD+MH from 2015 to 2020, were included in the study group; the MH was noted either preoperatively or discovered at the time of vitrectomy to repair the RRD. The diagnosis of MH was established either by preoperative OCT or intraoperative biomicroscopy. The diagnosis of macula hole could be confirmed easily with the use of dye in suspected cases. Another consecutive eyes with twice the number of the study group showing macula off RRD without concomitant macular hole during the same period served as the control group. Exclusion criteria were patients with preexisting MH noted before the acute symptoms of RRD, or preexisting retinal disease including proliferative diabetic retinopathy, retinal vascular occlusive disease, age related macular degeneration, etc., which would affect the macular status, and a clinical follow up of less than 6 months. Patients’ data were recorded, including initial and final best corrected visual acuity (BCVA) in Snellen chart, presence of choroidal detachment (CD) noted preoperatively or during the operation, extent of RRD, presence of proliferative vitreoretinopathy (PVR), and duration of RRD before surgical intervention. Axial length was measured when the retina was reattached with optical biometry (Lenstar LS 900, Haag–Streit, USA). Axial length longer or equal to 26 mm were defined as high myopia. RRD involved equal or more than three quadrants was defined as subtotal retinal detachment. Spectral domain optical coherence tomography (Spectralis OCT, Heidelberg Engineering, Germany in CCH and Optovue, USA, in NTUH) pre- and post-operatively at each visit was obtained. Closure of MH was evaluated with OCT. Surgical procedures including vitrectomy, air-fluid exchange and gas or silicone oil tamponade were recorded. Eyes in the group of RRD+MH may have membrane peeling, internal limiting membrane (ILM) peeling with or without ILM flap during the primary surgery. Eyes with PVR had extensive posterior ILM peeling during the primary surgery.

### Statistical analysis

Statistical analyses were performed using SPSS V.23.0. Visual acuity measurements were converted to logarithm of the minimum angle of resolution (logMAR) units for analysis. Mann–Whitney U test was used to compare the difference of age, pre-operative intraocular pressure, pre- and post-operative BCVA in logMAR, duration of retinal detachment between eyes with or without concomitant MH. Chi-square test was used to compare the difference of sex, presence of choroidal detachment (CD), total or subtotal RRD, recurrent RRD, high myopia and PVR between the two groups. For the eyes in the group of RRD+MH, univariate and multivariate regression analysis was used to find the correlation of various factors and final BCVA.

### Ethical approval

Since data were evaluated retrospectively, pseudonymously, and was solely obtained for treatment purposes, a requirement of informed consent was waived by the Ethics Committee of the Changhua Christian Hospital, Taiwan (IRB number 210421).

## Results

There were 40 eyes had RRD+MH with peripheral retinal breaks were included, among which, MH was noted preoperatively in 29 eyes and intraoperatively in 11 eyes. Another 80 consecutive eyes of RRD without MH noted either pre- or intra-operatively were served as the control group. Silicone oil tamponade was used in 16(40%) cases in RRD+MH group and 36(45%) cases in RRD without MH group. No difference regarding to age and sex, and duration of RD between these two groups were noted; however, eyes in the group of RRD+MH had worse initial, final VA at 6 months, and lower preoperative intraocular pressure. Besides, more eyes with RRD+MH had PVR, CD, total or subtotal RRD, and recurrent RD (Table 1). The use of silicone oil did not affect the surgical outcome.

**Table 1:** Comparison of demographic data between eyes of rhegmatogenous retinal detachment with or without concomitant macular hole.

	MHRD	RRD only	p
Age (years)	54.38 ± 9.50	54.11 ± 11.40	0.969*
Sex (M:F)	31:9	53:27	0.317^#^
Pre-op IOP (mmHg)	11.30 ± 4.70	13.32 ± 3.13	0.024*
Initial BCVA (logMAR)	2.48 ± 0.61	1.12 ± 0.96	< 0.001*
Total or subtotal RD (%)	30 (75%)	16 (20%)	< 0.001^#^
Recurrent RD (%)	9 (22.5%)	7 (8.75%)	0.013^#^
Combine PVR (%)	16 (40%)	10 (12.5%)	< 0.001^#^
Combine CD (%)	9 (22.5%)	1 (1.25%)	< 0.001^#^
Duration of RD ( months)	0.8 ± 2.94	0.21 ± 0.67	0.158*
High myopia (%)	10 (25%)	15 (18.75%)	0.366^#^
6 M BCVA (logMAR)	1.34 ± 0.57	0.40 ± 0.41	< 0.001*
Final BCVA (logMAR)	1.21 ± 0.75	0.27 ± 0.33	< 0.001*
Follow up duration (month)	21.59 ± 19.99	18.89 ± 7.96	0.372*

*M* male, *F* female, *IOP* intraocular pressure, *BCVA* best corrected visual acuity, *logMAR* logarithm of minimal angle of resolution, *MHRD* rhegmatogenous retinal detachment with concomitant macular hole, *RRD* rhegmatogenous retinal detachment, *PVR* proliferative vitreoretinopathy, *CD* choroidal detachment, *group 1* eyes with concomitant macular hole, *group 2* eyes without concomitant macular hole.

*Mann–Whitney U test.

#Chi-square test.

**Figure 1:**
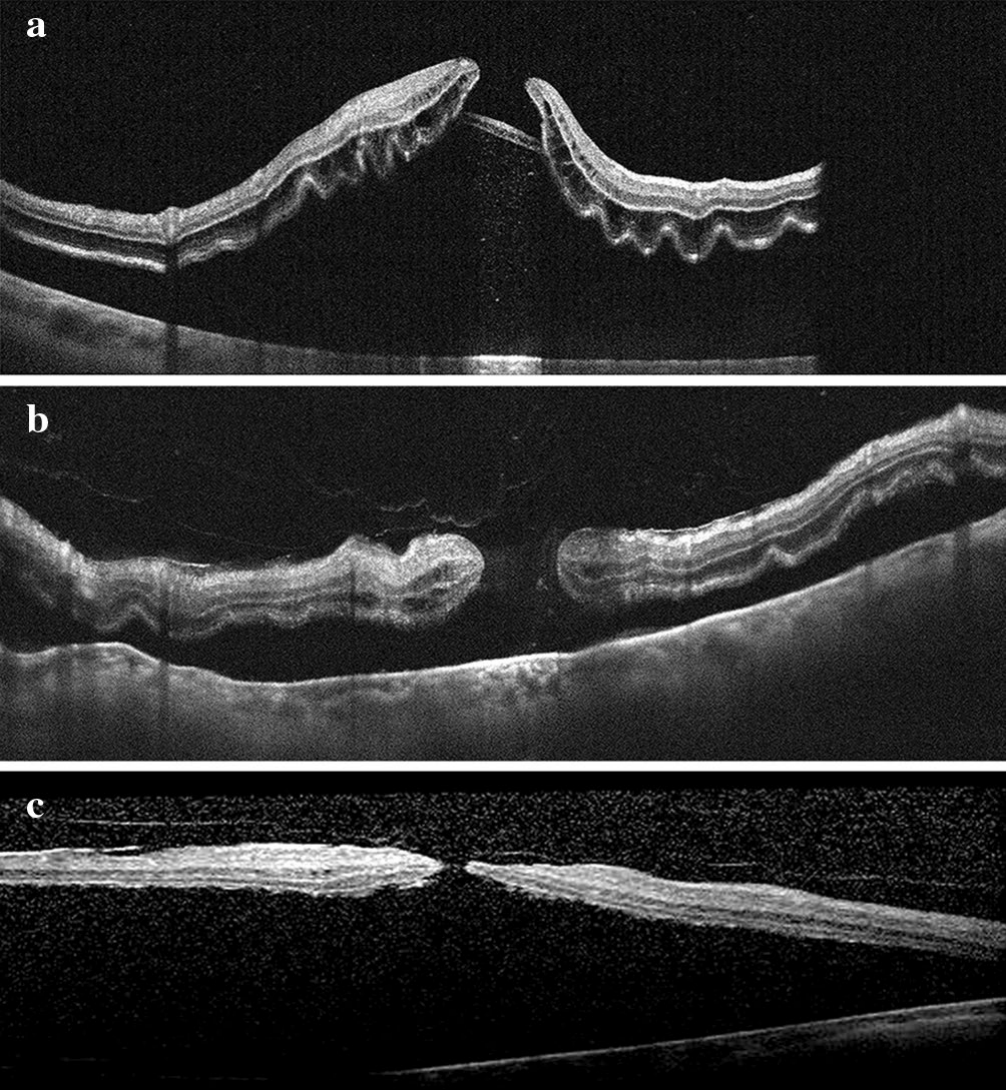
(**A**) A 49 year old male patient with concomitant macular hole and retinal detachment had ellipsoid zone bridging (the inner of the two lines) over the bottom of macular hole. Marked outer retina edema and corrugation was also noted. (**B**) A 59 year old male patient with concomitant retinal detachment and macular hole had macular epiretinal membrane noted. The outer retinal edema and corrugation was noted, but of less extent as compared to that of Fig. 1A. (**C**) A 50 year old female patient with concomitant retinal detachment and macular hole. Preoperative optical coherence tomography (OCT) showed incomplete posterior vitreous detachment, macular hole with thin rim, almost no retina edema was noted.

In the group of RRD+MH, preoperative OCT across the macular area was available in 17 eyes. For the other eyes, the preoperative OCT could not be obtained either due to vitreous hemorrhage (three eyes), or bullous retinal detachment (12 eyes). Among the 17 eyes with available pre-operative OCT, 11 eyes had ellipsoid zone bridging over the hole (Fig. 1A), and marked outer retinal corrugation was noted in all the 11 eyes; for the other six eyes, five eyes had epiretinal membrane present (Fig. 1B); one eye had epiretinal membrane and incomplete posterior vitreous detachment with thin fovea (Fig. 1C). Marked outer retinal corrugation was observed in only one of the six eyes.

In the group with RRD+MH, during the primary surgery, ILM peeling was performed in 19 eyes, in which 17 eyes had MH closed; ILM peeling along with hinged single layer flap were performed in 14 eyes, in which 11 eyes had MH closed, two eyes had flap displacement with MH unsealed and one eye had flat open configuration; the other seven eyes had neither ILM peeling nor ILM flap during primary surgery, and five eyes had MH open postoperatively. Repeated surgery was performed in the five eyes without primary ILM intervention and two of the two eyes with flap displacement, five of the seven eyes had MH closed after surgery. The hole closure rate at final follow up is 87.5%.

Multivariate regression analysis showed that initial BCVA, recurrent RRD, duration of RRD and the presence of PVR were correlated with poor final BCVA in the group of RRD+MH. (Table 2).

**Table 2:** Univariate and multivariate regression analysis for final best corrected visual acuity in eyes of rhegmatogenous retinal detachment with concomitant macular hole.

	Univariate regression analysis			Multivariate regression analysis		
	Beta	p	95% CI for beta	Beta	p	95% CI for beta
Initial BCVA (logMAR)	0.438	0.005*	0.183 to 0.938	0.895	< 0.001	0.637 to 0.837
Recurrent RD	− 0.179	0.275	− 0.915 to 0.268	− 0.157	0.004	− 0.661 to − 0.126
Combined CD	0.026	0.875	− 0.554 to 0.649	− 0.076	0.176	− 0.575 to 0.106
Duration of RD (month)	− 1.5	0.106	− 0.163 to 0.016	− 0.144	0.021	− 0.576 to − 0.048
PVR	− 0.352	0.026*	− 1.029 to − 0.069	− 0.171	0.001	− 0.131 to − 0.035
High myopia	0.08	0.629	− 0.432 to 0.704	− 0.036	0.48	− 0.291 to 0.137
RD range (total+subtotal v.s. partial)	0.223	0.166	− 0.171 to 0.96	0.024	0.734	− 0.138 to 0.195

*BCVA* best corrected visual acuity, *logMAR* logarithm of minimal angle of resolution, *RD* rhegmatogenous retinal detachment, *CD* choroidal detachment, *PVR* proliferative vitreoretinopathy.

*Mann-Whitney U test.

## Discussion

MH coexisting with RRD with peripheral causative breaks was uncommonly noted. It is different from MH induced RRD in high myopia, in which MH is the causative break for RRD. In contrast, in eyes of MH coexistent with RRD, the retina could be well attached by only addressing the peripheral causative breaks, either with scleral buckle or vitrectomy. Before the introduction of ILM peeling, the closure rate of macular hole was around 30% after vitrectomy and air-fluid exchange. With the technique of ILM peeling, about 80% of eyes could achieve MH closure.

According to previous reports, most eyes of RRD+MH were macula off^1,2,4,5^. Since macula attachment status would affect the pre- and postoperative visual outcome, to make the comparison more meaningful, we only include eyes with macula off RRD both in the study group and control group.

**Figure 2:**
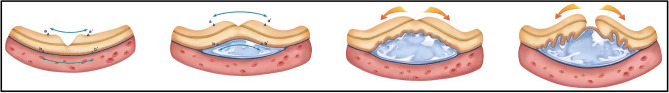
Schematic illustration of the possible evolution of macular hole in eyes with rhegmatogenous retinal detachment. From left to right: in attached retina, the a to a’ distance is shorter than b to b’. When retina detaches at macular area, the retina curvature changes from concave to convex shape, thus the distance from a to a’ becomes larger than b to b’. As the retinal detachment increases in height, the tangential force of inner retina increases which makes a break at fovea from inner retinal surface. In contrast, the less tangential force, outer retinal edema and corrugation make the outer retina relatively more redundant and leaves the ellipsoid zone at fovea.

The pathogenesis of MH concomitant with macular off RRD is still unknown. Some authors believed that the acute posterior vitreous detachment process, which was responsible for the formation of peripheral retinal breaks and hence the RD, also contributes to the formation of the full-thickness macular hole^3^. Some authors believe that tangential traction by the pre-retinal membrane may contribute to the development of MH^1^, which could be supported by the high incidence of PVR and presence of ERM in five of our patients with pre-operative OCT. The tangential traction on fovea from epiretinal membrane may further be aggravated by the stretching force of retinal detachment which induce an inward bulging of macula^6^. Other possible pathogenesis including foveal cystoid changes secondary to hypoxia and inflammatory changes induced by neuroretinal detachment, which weaken the fovea structure. In this study, we noticed that most of the MH in RRD+MH had EZ lining the bottom of macular hole. This possible explanation would be that an attached retina is in a concave shape and has the inner neuroretinal surface smaller than the outer neuroretinal surface. When the retina is detached, the retina becomes convex in shape, thus the inner neuroretinal surface area expands and suffers from tangential traction, in contrast, the outer retina becomes concave and the surface are relatively more redundant than the inner retinal surface. Besides, the hypoxia induced outer retina edema and secondary corrugation, which was observed in all eyes of EZ lining in our cases, may further increase the redundancy. (Fig. 2) The difference of geometry and structural changes of detached retina may well explain the breaking point of MH would start from the inner surface with the ellipsoid zone still bridging the fovea as shown in most of the preoperative OCT in our series, which is different from the idiopathic macular hole from anterior–posterior traction, that the outer retinal defect generally precedes the inner retina defect^7,8^.

In the current study, we noted that eyes of RRD+MH had higher chance of PVR, CD, more extensive RRD and recurrent RRD. Poor initial and final BCVA in eyes with concomitant MH than eyes without MH had also been reported in previous literature, even though the holes were successfully repaired^5^. The poor visual outcome may be explained by that the presence of MH itself has negative impact on vision recovery, besides, the possible different pathogenesis of MH in RRD+MH including cystoid macular changes, which may cause permanent tissue damage and further aggravate the vision recovery. PVR has been reported of higher incidence^1,2^ and as a risk factor for macular hole eyes with RRD^2^. The presence of PVR surface membranes is very likely to induce additional tangential forces, thus may play a role in the simultaneous occurrence of a MH and RRD.

Previous reports have shown that MH was a risk factor for CD^9–11^. In this study, we further showed that the incidence of CD is significantly higher in eyes with concomitant MH. Presence of CD would markedly increase intraocular inflammatory cytokine^12^, which may aggravate the cystoid changes of the already compromise macula, and the development of PVR^13^, thus facilitate the formation of MH. More extensive RRD was also noted in our series of RRD+MH. Previous report^1^ had also shown a high incidence of subtotal or total RRD (91%) in eyes of RRD+MH. Eyes of RRD had been shown to have elevated inflammatory and ischemic cytokines including VEGF, IL-8 in the vitreous^14^. Besides, elevated levels of tumor necrosis factor α (TNF-α), monocyte chemotactic protein-1 (MCP-1), and basic fibroblast growth factor (bFGF) were also detected in detached neural retina^15^. A more extensive retinal detachment may induce more cytokine release, with more severe secondary cystoid changes and retina apoptosis, which may also help the formation of MH. The current study showed increased incidence of recurrent RRD in eyes with concomitant MH, which is in line with the previous report^2^, and was within the expectation, as patients in this group had higher chance of PVR, CD, and extensive RRD, all of which would increase the incidence of recurrent RRD^16^. Echos with previous studies, our study showed that eyes of MH had worse initial and final BCVA, which is fairly reasonable, as MH, PVR, extensive RRD, and recurrent RRD all contributed to poor initial and final BCVA.

The limitations of this study include the retrospective nature and lack of intraoperative and preoperative OCT in most cases; as a thin fovea surrounded by edematous retina during intraoperative observation may give the false impression of a macular hole, though the Schlieren phenomenon, and application of dye may easily tell the presence of MH. The difference between surgeons may also confound the analysis. With the more widely use of intraoperative OCT in the future, the presence of MH maybe more confirmative and the morphology of MH concomitant with RRD could be more thoroughly appreciated in eyes without preoperative OCT. Besides, though we try to exclude eyes with preexisting MH before the event of RRD by tracing back the patients’ history, it is possible that some of our patients had MH previous to this event of RRD.

In conclusion, patients with RRD+MH had both worse initial and final visual acuity, higher incidence of PVR and CD, more extensive RRD and higher chance of recurrent RD. Besides, most of the MH obtained preoperatively had ellipsoid zone bridging the bottom of MH, cystoid macular changes and changing of retinal curvature from submacular fluid may be one of the major pathogenic factors for MH formation.

## Data Availability

The datasets generated and/or analysed during the current study are not publicly available but are available from the corresponding author on reasonable request.
